# Phosphorylation and Proteasome Recognition of the mRNA-Binding Protein Cth2 Facilitates Yeast Adaptation to Iron Deficiency

**DOI:** 10.1128/mBio.01694-18

**Published:** 2018-09-18

**Authors:** Antonia M. Romero, Mar Martínez-Pastor, Gang Du, Carme Solé, María Carlos, Sandra V. Vergara, Nerea Sanvisens, James A. Wohlschlegel, David P. Toczyski, Francesc Posas, Eulàlia de Nadal, María T. Martínez-Pastor, Dennis J. Thiele, Sergi Puig

**Affiliations:** a Departamento de Biotecnología, Instituto de Agroquímica y Tecnología de Alimentos (IATA), Consejo Superior de Investigaciones Científicas (CSIC), Paterna, Valencia, Spain; b Department of Pharmacology and Cancer Biology, Duke University School of Medicine, Durham, North Carolina; c Department of Biochemistry, Duke University School of Medicine, Durham, North Carolina, USA; d Department of Molecular Genetics and Microbiology, Duke University School of Medicine, Durham, North Carolina, USA; e Departamento de Ciències Experimentals i de la Salut, Cell Signaling Research Group, Universitat Pompeu Fabra, Barcelona, Spain; f Tianjin Key Laboratory of Food Biotechnology, College of Biotechnology and Food Science, Tianjin University of Commerce, Tianjin, China; g Department of Biochemistry and Biophysics, Helen Diller Family Comprehensive Cancer Center, University of California, San Francisco, California, USA; h Department of Biological Chemistry, University of California, Los Angeles, California, USA; i Departamento de Bioquímica y Biología Molecular, Universitat de València, Burjassot, Valencia, Spain; National Institutes of Health

**Keywords:** iron deficiency, phosphorylation, posttranslational regulation, protein stability, yeast

## Abstract

Iron is an indispensable micronutrient for all eukaryotic organisms due to its participation as a redox cofactor in many metabolic pathways. Iron imbalance leads to the most frequent human nutritional deficiency in the world. Adaptation to iron limitation requires a global reorganization of the cellular metabolism directed to prioritize iron utilization for essential processes. In response to iron scarcity, the conserved Saccharomyces cerevisiae mRNA-binding protein Cth2, which belongs to the tristetraprolin family of tandem zinc finger proteins, coordinates a global remodeling of the cellular metabolism by promoting the degradation of multiple mRNAs encoding highly iron-consuming proteins. In this work, we identify a critical mechanism for the degradation of Cth2 protein during the adaptation to iron deficiency. Phosphorylation of a patch of Cth2 serine residues within its amino-terminal region facilitates recognition by the SCF^Grr1^ ubiquitin ligase complex, accelerating Cth2 turnover by the proteasome. When Cth2 degradation is impaired by either mutagenesis of the Cth2 serine residues or deletion of *GRR1*, the levels of Cth2 rise and abrogate growth in iron-depleted conditions. Finally, we uncover that the casein kinase Hrr25 phosphorylates and promotes Cth2 destabilization. These results reveal a sophisticated posttranslational regulatory pathway necessary for the adaptation to iron depletion.

## Abstract (precis)

Iron is a vital element for many metabolic pathways, including the synthesis of DNA and proteins, and the generation of energy via oxidative phosphorylation. Therefore, living organisms have developed tightly controlled mechanisms to properly distribute iron, since imbalances lead to nutritional deficiencies, multiple diseases, and vulnerability against pathogens. Saccharomyces cerevisiae Cth2 is a conserved mRNA-binding protein that coordinates a global reprogramming of iron metabolism in response to iron deficiency in order to optimize its utilization. Here we report that the phosphorylation of Cth2 at specific serine residues is essential to regulate the stability of the protein and adaptation to iron depletion. We identify the kinase and ubiquitination machinery implicated in this process to establish a posttranscriptional regulatory model. These results and recent findings for both mammals and plants reinforce the privileged position of E3 ubiquitin ligases and phosphorylation events in the regulation of eukaryotic iron homeostasis.

## INTRODUCTION

Iron is an essential micronutrient for all eukaryotic organisms because it participates as a catalytic redox cofactor in numerous metabolic pathways, including energy generation through mitochondrial respiration, protein translation, DNA replication and repair, and lipid biosynthesis. Despite being very abundant, iron bioavailability is highly restricted due to its low solubility at physiological pH. Indeed, iron deficiency anemia represents the most common nutritional disorder in humans, thought to affect more than two billion people, with large impact on pregnant women and children (1). Moreover, iron imbalances lead to both frequent and rare human diseases, including hereditary hemochromatosis, Friedreich’s ataxia, and aceruloplasminemia, and influence fungal and bacterial pathogenesis (2). Therefore, eukaryotic cells have developed sophisticated molecular mechanisms to properly respond to alterations in iron levels.

In response to iron deficiency, the budding yeast Saccharomyces cerevisiae activates the expression of Cth2, an mRNA-binding protein that, in coordination with its partially redundant homolog Cth1, plays a pivotal role in the adaptation to iron limitation by posttranscriptionally remodeling iron metabolism (3, 4). Cth1 and Cth2 belong to a family of proteins conserved in all eukaryotes that is characterized by the presence of two tandem zinc fingers (TZFs) of the CX_8_CX_5_CX_3_H type, which directly interact with AU-rich elements (AREs) located in the 3′-untranslated region (3′-UTR) of many mRNAs to promote their rapid destabilization and inhibit their translation (3–6). Humans express three TZF-containing proteins, with tristetraprolin being the most studied family member (reviewed in references 7 and 8). Upon iron scarcity, Cth1 and Cth2 coordinately promote the decay of mRNAs that encode proteins with functions in highly iron-consuming pathways, including the mitochondrial electron transport chain and the tricarboxylic acid cycle, to prioritize its utilization in essential iron-dependent processes such as deoxyribonucleotide synthesis by ribonucleotide reductase (3, 4, 9). Cth2 binds to its target transcripts in the nucleus. Then, the mRNA-Cth2 complexes are exported to the cytoplasm where mRNA turnover and translation inhibition take place (6, 10, 11). Mutagenesis of a conserved cysteine residue within Cth2 TZFs abrogates both its interaction with ARE-containing mRNAs and its nuclear export (3, 11). Cells lacking a functional Cth2 protein (*cth2*Δ cells or Cth2-TZF mutants) grow properly under iron-replete conditions but display growth impairment under iron-deficient conditions due to defects in the downregulation of multiple iron-related mRNAs (3, 6). Although the molecular reasons are not known, the overexpression of a functional Cth2 protein under iron-sufficient conditions is also detrimental for growth (5, 10). Therefore, yeast cells have developed refined strategies to tightly control Cth2 expression levels to satisfy cellular iron demands. First, the partially redundant Aft1 and Aft2 iron-responsive factors specifically activate *CTH2* transcription in response to iron deprivation, whereas Cth2 protein is not present under iron-sufficient conditions (3). Second, the *CTH2* mRNA contains an ARE within its 3′-UTR that limits its own expression through a negative-feedback autoregulatory mechanism (6, 12).

In this study, we identified key amino acid residues of the TZF-containing protein Cth2 that are phosphorylated by the Hrr25 kinase. These phosphorylation events facilitate Cth2 recognition by the SCF^Grr1^ ubiquitin ligase complex, which promotes its proteasomal degradation. This posttranslational regulatory process is essential for the adaptation of yeast cells to iron deficiency.

## RESULTS

### The yeast Cth2 protein is phosphorylated at specific serine residues.

**FIG 1:**
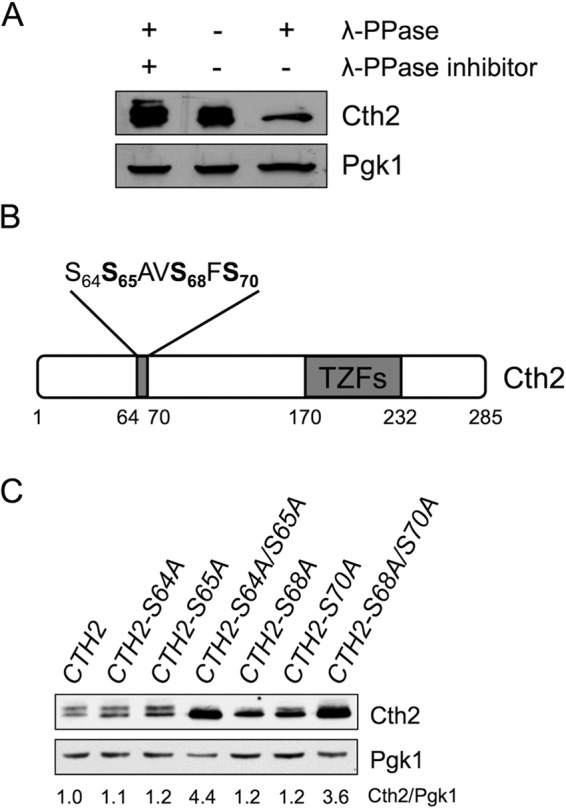
Cth2 protein is phosphorylated at specific serine residues. (A) Cth2 is a phosphoprotein. Yeast *cth2*Δ (SPY25) cells transformed with pRS416-Flag_2_-Cth2 were grown to exponential phase in synthetic complete medium without uracil (SC-ura) with 100 μM BPS. Protein extracts were treated with λ phosphatase (λ-PPase) in the presence (+) or absence (−) of protein phosphatase inhibitors: 66 mM NaF and 66 mM EDTA. (B) Schematic representation of Cth2 phosphorylated residues. Flag_2_-Cth2 immunoprecipitation and mass spectrometry indicate that Cth2 S65, S68, and S70 are phosphorylated *in vivo*. Cth2 TZFs are represented. Numbers refer to amino acid positions. (C) Electrophoretic mobility and relative abundance of wild-type and Cth2 proteins mutagenized at specific serine residues. Yeast *cth1*Δ *cth2*Δ (SPY122) cells transformed with plasmid pRS416-Flag_2_-Cth2, pRS416-Flag_2_-Cth2-S64A, pRS416-Flag_2_-Cth2-S65A, pRS416-Flag_2_-Cth2-S64A/S65A, pRS416-Flag_2_-Cth2-S68A, pRS416-Flag_2_-Cth2-S70A, or pRS416-Flag_2_-Cth2-S68A/S70A were grown in SC-ura with 100 μM BPS. Total proteins were extracted, and Cth2 and Pgk1 protein levels were determined by immunoblotting with anti-Flag and anti-Pgk1 antibodies, respectively. Pgk1 was used as a loading control. Cth2/Pgk1 protein quantitation is shown relative to the values obtained for cells expressing wild-type Cth2.

In response to iron deficiency, the budding yeast S. cerevisiae expresses two iron-regulated TZF proteins named Cth1 and Cth2 (3–5). When we analyzed the electrophoretic mobility of functional, Flag epitope-tagged Cth1 and Cth2 proteins on SDS-polyacrylamide gels, we observed a slow-migrating band in addition to the bands expected according to their size (3, 12) (Fig. 1A; see also Fig. S1 in the supplemental material). To ascertain whether the altered electrophoretic mobility was due to phosphorylation, we treated the protein extracts from yeast cells with λ phosphatase. We observed that the upper band disappeared when the extracts were incubated with λ phosphatase, but it was still present when λ phosphatase inhibitors were included in the assay (Fig. 1A and Fig. S1). These results strongly suggest that both Cth1 and Cth2 are phosphoproteins.

10.1128/mBio.01694-18.1 FIG S1 Yeast Cth1 is a phosphoprotein. (A) Yeast *cth1*Δ (SPY131) cells transformed with pRS416-Flag_2_-Cth1 plasmid were grown and proteins were analyzed as described in the legend of Fig. 1A. (B) Cth1 and Cth2 protein alignment. The sequence corresponding to the TZFs is shown in red, whereas the Cth1 serine residues conserved in the Cth2-S64/S65/S68/S70 patch are indicated in blue. Asterisks indicate conserved residues. (C) Schematic representation of Cth1 conserved serine residues within the Cth2-S64/S65/S68/S70 patch. Numbers refer to amino acid positions. Download FIG S1, PDF file, 0.1 MB. Copyright © 2018 Romero et al. 2018 Romero et al. This content is distributed under the terms of the Creative Commons Attribution 4.0 International license.

We focused our studies on Cth2 protein because of its predominant role over Cth1 in the adaptation of yeast cells to iron depletion (3, 4). To decipher the amino acids specifically phosphorylated on Cth2, we grew cells expressing Flag_2_-Cth2 (Cth2 tagged with two copies of the Flag epitope) under iron-deficient conditions, achieved by the addition of the Fe^2+^-specific chelator bathophenanthroline disulfonic acid (BPS), and we specifically immunoprecipitated Cth2 protein. Mass spectrometry revealed that Cth2 serine residues S65, S68, and S70 were phosphorylated, with a predominance of peptides with phosphorylated S68 (schematic representation in Fig. 1B). Interestingly, Cth2 S65 and S68 residues are conserved in Cth1 protein (Fig. S1). To further ascertain the relevance of these serine residues in Cth2 phosphorylation, we mutagenized them and the nearby S64 residue to alanine. An immunoblot analysis showed that single S64A and S65A mutations did not change the electrophoretic pattern of Cth2, the S70A substitution only partially decreased Cth2 phosphorylation, and mutagenesis of S68 almost fully collapsed Cth2 phosphorylation shift (Fig. 1C). Importantly, the Cth2-S64A/S65A, Cth2-S68A/S70A, and Cth2-S64A/S65A/S68A/S70A mutations abolished most Cth2 phosphorylation and increased Cth2 protein abundance to 1.5- to 4.4-fold (Fig. 1C and Fig. S2A). These results suggest that all four serine residues analyzed are phosphorylated *in vivo*.

**FIG 2:**
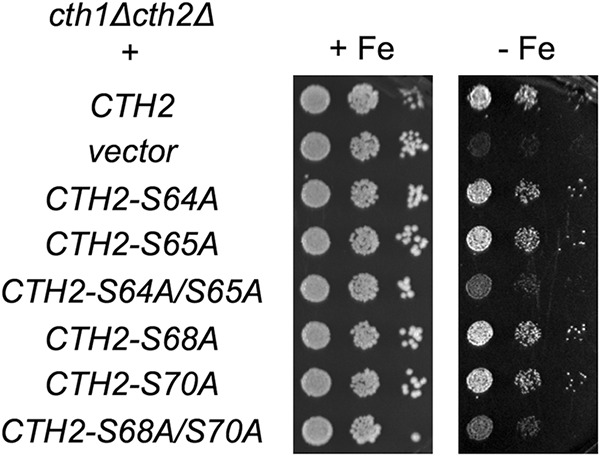
The Cth2 serine residues S64 and S65 (S64/S65) and S68/S70 are required for adaptation to iron-deficient conditions. *cth1*Δ *cth2*Δ (SPY122) strain was transformed with plasmid pRS416-Flag_2_-Cth2, pRS416 (vector), pRS416-Flag_2_-Cth2-S64A, pRS416-Flag_2_-Cth2-S65A, pRS416-Flag_2_-Cth2-S64A/S65A, pRS416-Flag_2_-Cth2-S68A, pRS416-Flag_2_-Cth2-S70A, or pRS416-Flag_2_-Cth2-S68A/S70A. Yeast transformants were grown in liquid SC-ura to exponential phase and spotted in 10-fold serial dilutions on SC-ura (with Fe [+Fe]) and SC-ura containing 900 μM Ferrozine (without Fe [−Fe]). Two biological replicates were incubated at 30°C for 3 days (+Fe) or for a week (−Fe) and then photographed.

**FIG 3:**
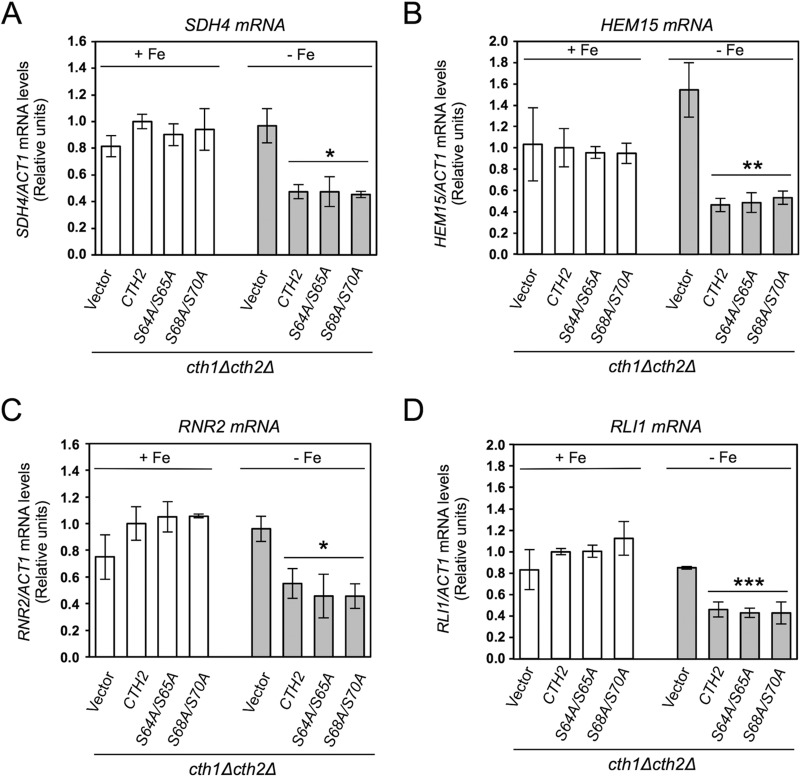
Cth2-S64A/S65A and Cth2-S68A/S70A mutants function in targeted mRNA degradation. *cth1*Δ *cth2*Δ (SPY122) cells transformed with plasmid pRS416-Flag_2_-Cth2, pRS416 (empty vector), pRS416-Flag_2_-Cth2-S64A/S65A, or pRS416-Flag_2_-Cth2-S68A/S70A were grown in SC-ura (+Fe; white bars) or SC-ura with 100 μM BPS (−Fe; gray bars). (A to D) Total RNA was extracted, and the *SDH4* (A), *HEM15* (B), *RNR2* (C), *RLI1* (D), and *ACT1* mRNA levels were determined by RT-qPCR. *ACT1* was used to normalize the mRNA values. Values are shown relative to the values for *cth1*Δ *cth2*Δ cells expressing wild-type *CTH2* with Fe. The means ± standard deviations (error bars) for three independent biological experiments are shown. No significant differences were observed between cells containing the empty vector grown under conditions in the presence and absence of Fe. Values that are statistically significantly different for yeast cells grown without Fe compared to those grown with Fe are indicated by asterisks as follows: *, *P* < 0.03; **, *P* < 0.01; ***, *P* < 0.004.

10.1128/mBio.01694-18.2 FIG S2 Characterization of the Cth2-S64A/S65A/S68A/S70A mutant. (A) Electrophoretic mobility and relative abundance of Cth2-S64A/S65A/S68A/S70A mutant protein. Yeast *cth1*Δ *cth2*Δ (SPY122) cells containing pRS416-Flag_2_-Cth2, pRS416-Flag_2_-Cth2-S64A/S65A, pRS416-Flag_2_-Cth2-S68A/S70A, or pRS416-Flag_2_-Cth2-S64A/S65A/S68A/S70A plasmid were grown and analyzed as described in the legend of Fig. 1C. (B) Growth of yeast cells expressing *CTH2-S64A/S65A/S68A/S70A* under iron-deficient conditions. *cth1*Δ *cth2*Δ (SPY122) yeast cells transformed with pRS416-Flag_2_-Cth2, pRS416-Flag_2_-Cth2-S64A/S65A, pRS416-Flag_2_-Cth2-S68A/S70A, and pRS416-Flag_2_-Cth2-S64A/S65A/S68A/S70A plasmids were assayed as described in the legend of Fig. 2. Ferrozine (−Fe) was added at 600 and 700 μM concentrations. (C) Cth2-S64A/S65A/S68A/S70A mutant functions in targeted mRNA degradation. *cth1*Δ *cth2*Δ (SPY122) yeast cells transformed with pRS416-Flag_2_-Cth2-S64A/S65A/S68A/S70A plasmid were grown in SC-ura (+Fe; white bars) or SC-ura with 100 μM BPS (−Fe; gray bars). Total RNA was extracted and analyzed as indicated in the legend of Fig. 3. Values are shown relative to the values for +Fe cells. The averages from two independent biological experiments are shown. (D) Determination of Cth2-S64A/S65A/S68A/S70A protein stability. *cth1*Δ *cth2*Δ (SPY122) yeast cells transformed with pRS416-Flag_2_-Cth2 or pRS416-Flag_2_-Cth2-S64A/S65A/S68A/S70A plasmid were grown and analyzed as shown in the legend of Fig. 5. Cth2-S64A/S65A/S68A/S70A protein half-life was calculated on the basis of two independent biological experiments. The average and standard deviation are shown. Download FIG S2, PDF file, 0.2 MB. Copyright © 2018 Romero et al. 2018 Romero et al. This content is distributed under the terms of the Creative Commons Attribution 4.0 International license.

### Cth2 phosphorylation is required for growth under iron starvation.

To determine the physiological relevance of Cth2 phosphorylation in the response of yeast cells to iron deprivation, we expressed Cth2 serine mutants in a *cth1*Δ *cth2*Δ double mutant strain (to eliminate any *CTH1* redundancy) and assayed growth in iron-deficient conditions, achieved in this case by the addition of the Fe^2+^-specific chelator Ferrozine. We observed that the single Cth2 serine mutants did not display any significant growth defect under iron-limiting conditions compared to cells expressing wild-type Cth2 (Fig. 2). Remarkably, both Cth2-S64A/S65A and Cth2-S68A/S70A double mutants exhibited a growth defect under low-iron conditions (Fig. 2). Strikingly, the Cth2-S64A/S65A/S68A/S70A quadruple mutant partially rescued the growth defect under iron-deficient conditions observed for the Cth2 double mutants (Fig. S2B). These results indicate that both Cth2-S64/S65 and Cth2-S68/S70 serine pairs are important for optimal adaptation to iron deficiency.

### The Cth2 serine mutants function in targeted mRNA degradation.

To determine whether Cth2 serine mutants had defects in targeted mRNA degradation under conditions of iron scarcity, we determined the mRNA levels of various Cth2 target transcripts in cells grown in both iron-sufficient and iron-deficient media. In particular, we determined the mRNA levels for *SDH4*, which encodes a subunit of succinate dehydrogenase in the tricarboxylic acid cycle; *HEM15*, which encodes ferrochelatase, the last step in the heme biosynthetic pathway; *RNR2*, a small subunit of ribonucleotide reductase in deoxyribonucleotide synthesis; and RNase L inhibitor *RLI1*, which is required for ribosome recycling during protein translation. Surprisingly, cells expressing *CTH2* serine mutants *S64A/S65A*, *S68A/S70A* or *S64A/S65A/S68A/S70A* promoted the downregulation of the four transcripts upon iron scarcity in a manner similar to that of wild-type *CTH2* (Fig. 3 and Fig. S2C). These results suggest that the growth defect displayed by the Cth2 serine mutants under iron-deficient conditions is not due to defects in targeted mRNA decay.

### The patch of S64/S65/S68/S70 residues facilitates Cth2 protein degradation.

**FIG 4:**
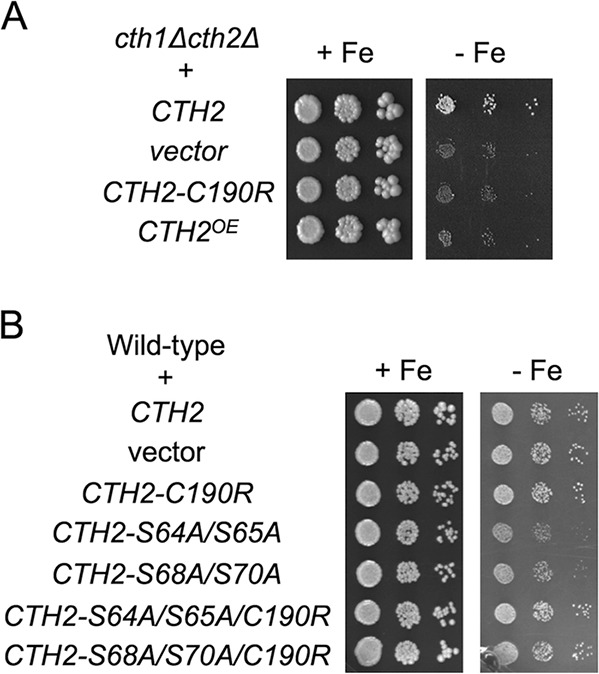
The expression of *CTH2* serine mutants in wild-type cells causes growth defects in iron-deficient conditions. (A) Both the lack of a functional Cth2 protein and *CTH2* overexpression are detrimental for adaptation to iron deficiency. The yeast *cth1*Δ *cth2*Δ (SPY122) strain was transformed with plasmid pRS416-Flag_2_-Cth2, pRS416 (vector), pRS416-Flag_2_-Cth2-C190R, or pRS426-Flag_2_-Cth2 (*CTH2*^OE^). (B) The expression of Cth2-S64A/S65A or Cth2-S68A/S70A is toxic for wild-type cells. The wild-type (BY4741) strain was transformed with pRS416-Flag_2_-Cth2, pRS416 (vector), pRS416-Flag_2_-Cth2-C190R, pRS416-Flag_2_-Cth2-S64A/S65A, pRS416-Flag_2_-Cth2-S68A/S70A, pRS416-Flag_2_-Cth2-S64A/S65A/C190R, or pRS416-Flag_2_-Cth2-S68A/S70A/C190R. In panels A and B, yeast transformants were grown in liquid SC-ura to exponential phase and spotted in 10-fold serial dilutions on SC-ura (+Fe) and SC-ura containing 700 μM Ferrozine (−Fe in panel A) or 900 μM Ferrozine (−Fe in panel B). Two biological replicates were incubated at 30°C for 3 days (+Fe) or a week (−Fe) and then photographed.

Previous studies have shown that overexpression of a functional TZF-containing Cth2 protein is cytotoxic for yeast cells (5, 10, 12). Indeed, both the lack of a functional Cth2 protein (*cth1*Δ *cth2*Δ cells containing either empty vector or the TZF mutant allele *CTH2-C190R*) or the overexpression of wild-type *CTH2* (*cth1*Δ *cth2*Δ cells with a multicopy *CTH2*^OE^ plasmid [where OE stands for overexpression]) led to similar growth defects under iron starvation conditions (Fig. 4A). Therefore, we considered whether the growth defect observed for the Cth2 serine mutants under iron-deficient conditions was due to their high protein levels (Fig. 1C). If this were the case, *CTH2-S64A/S65A* and *CTH2-S68A/S70A* would be gain-of-function alleles, and unlike the loss-of-function *CTH2-C190R* allele, these alleles should be toxic when expressed in wild-type cells. Consistent with this hypothesis, the expression of the Cth2 double serine mutants impaired the growth of wild-type cells in iron-deficient media, whereas *CTH2-C190R* expression did not alter growth (Fig. 4B). Furthermore, the growth defect triggered by the expression of Cth2 double serine mutants was abolished when the Cth2 TZFs were mutagenized, which indicates that Cth2-S64A/S65A and Cth2-S68A/S70A deleterious effects require binding to target mRNAs (Fig. 4B). As previously observed for *CTH2*^OE^ cells (10), these results are consistent with a gain of function for Cth2-S64A/S65A and Cth2-S68A/S70A proteins, which are unable to adapt to iron starvation.

**FIG 5:**
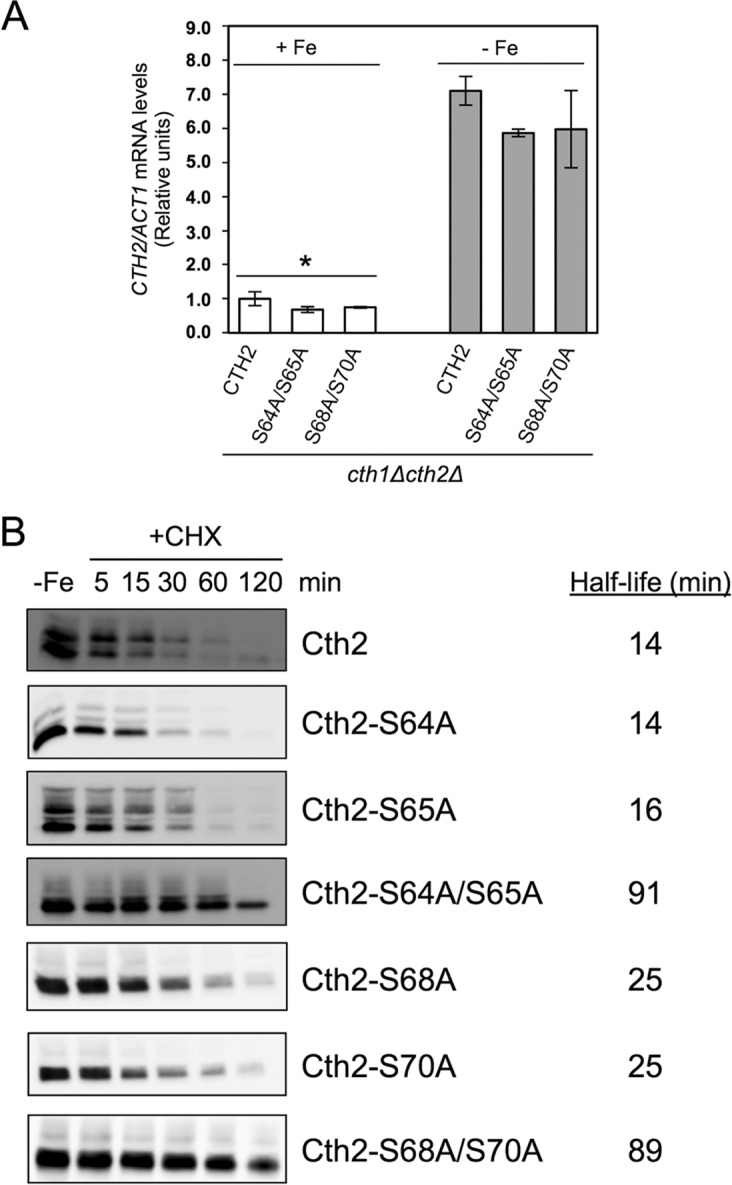
Mutagenesis of specific serine residues increases Cth2 protein stability. (A) *CTH2-S64A/S65A* and *CTH2-S68A/S70A* mRNA levels are similar to wild-type *CTH2*. *cth1*Δ *cth2*Δ (SPY122) cells transformed with plasmid pRS416-Flag_2_-Cth2, pRS416-Flag_2_-Cth2-S64A/S65A, or pRS416-Flag_2_-Cth2-S68A/S70A were grown in SC-ura (+Fe; white bars) or SC-ura with 100 μM BPS (−Fe; gray bars). Total RNA was extracted, and the *CTH2* and *ACT1* mRNA levels were determined by RT-qPCR. *ACT1* was used to normalize the mRNA values. Values are relative to the values for *cth1*Δ *cth2*Δ cells expressing wild-type *CTH2* with Fe. No significant differences were observed among cells expressing *CTH2*, *CTH2-S64A/S65A*, and *CTH2-S68A/S70A*. The means ± standard deviations for three independent biological experiments are shown. Values that are statistically significantly different (*P* < 0.03) in cells grown with Fe and cells grown without Fe are indicated by an asterisk. (B) Determination of the stability of different Cth2 proteins. *cth1*Δ *cth2*Δ (SPY122) yeast cells transformed as described in the legend of Fig. 1C were grown in SC-ura containing 100 μM BPS (−Fe). Then, 50 μg/ml cycloheximide (CHX) was added to stop translation, and aliquots were isolated at the indicated times. Total proteins were extracted, and Cth2 protein levels were determined by immunoblotting with anti-Flag antibody. Equal amounts of total proteins were loaded in each lane. Cth2 protein levels were determined, and the average half-life was calculated (*n* = 3 for Cth2-S64A/S65A and Cth2-S68A/S70A and *n* = 2 for the rest of experiments). A representative immunoblot is shown.

We then explored the molecular basis for the increase of abundance of the Cth2 serine mutant proteins. We first determined *CTH2-S64A/S65A* and *CTH2-S68A/S70A* transcript levels compared to wild-type *CTH2* mRNA under conditions of iron sufficiency and iron deficiency. We observed that the levels of *CTH2-S64A/S65A* and *CTH2-S68A/S70A* mRNAs increased upon iron depletion, but their abundance was not higher than that of the wild-type *CTH2* (Fig. 5A). These results indicated that the increased levels of Cth2 serine mutant proteins were not due to higher transcript abundance. Then, we ascertained whether the stability of these Cth2 serine mutant proteins was different from that of the wild-type protein. For this purpose, we grew yeast cells expressing the different Flag-tagged Cth2 proteins under iron-deficient conditions, stopped protein synthesis by the addition of cycloheximide (CHX), and determined the turnover rate of the Cth2 proteins. Our assays indicated that wild-type Cth2 protein had a half-life of approximately 14 min (Fig. 5B). A similar result was obtained when a functional amino-terminal Myc-tagged Cth2 protein was used in this assay (Fig. S3). Some of the single Cth2 serine mutants displayed a slight increase in protein stability (S65A, S68A, and S70A [Fig. 5B]). However, mutagenesis of S64/S65 or S68/S70 residues increased the half-life of the Cth2 protein approximately sixfold (from 14 to 90 min [Fig. 5B]). Interestingly, simultaneous mutagenesis of the four S64/S65/S68/S70 residues increased Cth2 protein stability only fourfold (from 14 to 60 min [Fig. S2D]). These results demonstrate that the S64/S65/S68/S70 residues determine the stability of Cth2 protein under low-iron conditions. Furthermore, they also indicate that both Cth2 S64/S65 and S68/S70 serine pairs are required to promote Cth2 protein turnover under iron-deficient conditions.

10.1128/mBio.01694-18.3 FIG S3 Determination of Myc2-Cth2 protein half-life. (A) *cth1*Δ *cth2*Δ (SPY122) cells transformed with pRS416-Myc_2_-Cth2 plasmid were grown and assayed as described in the legend of Fig. 5B. Total proteins were extracted, and Cth2 protein levels were determined by immunoblotting with anti-Myc antibody. Pgk1 was used as a loading control. (B) Quantitation of protein levels in panel A. Download FIG S3, PDF file, 0.2 MB. Copyright © 2018 Romero et al. 2018 Romero et al. This content is distributed under the terms of the Creative Commons Attribution 4.0 International license.

### The SCF^Grr1^ ubiquitin ligase complex facilitates Cth2 protein degradation by the proteasome.

**FIG 6:**
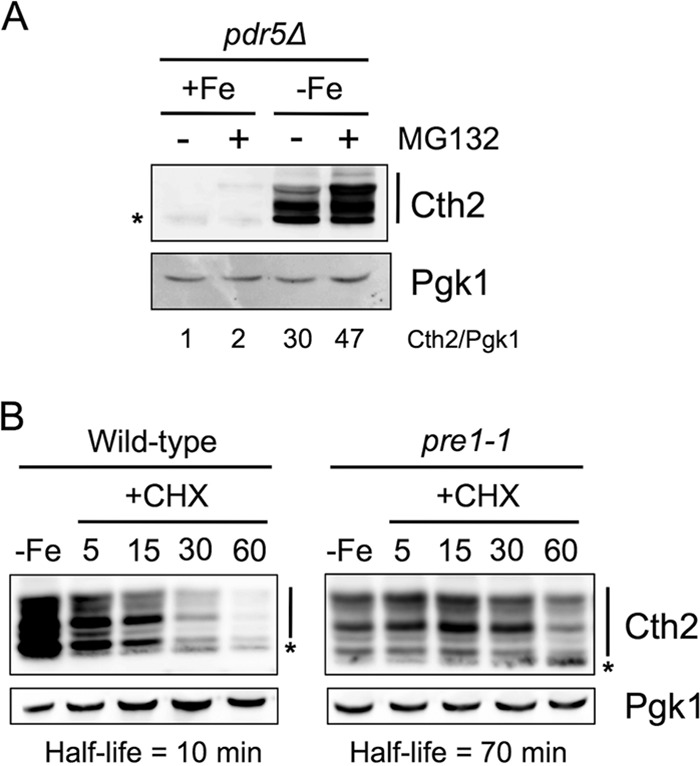
Cth2 protein is degraded by the proteasome. (A) MG132 inhibits Cth2 protein degradation. *pdr5*Δ (SPY970) cells transformed with pRS416-Flag_2_-Cth2 plasmid were grown in SC-ura (+Fe) or SC-ura with 100 μM BPS (−Fe) and treated with 100 μM MG132 for 60 min or with DMSO as a negative control. Protein was extracted, and Cth2 and Pgk1 protein levels were determined by immunoblotting. (B) Cells defective in the proteasome stabilize Cth2 protein. Wild-type (YWO0607) and *pre1-1* (YWO0608) yeast strains transformed with pRS416-Flag_2_-Cth2 plasmid were grown in SC medium for 3 h at 37°C. Then, 100 μM BPS was added, and the cells were incubated for 6 additional hours at 37°C (−Fe). At this point, cycloheximide (CHX) was added to stop translation, and aliquots were isolated and analyzed at the indicated times (in minutes). A representative experiment of two independent biological assays is shown. The asterisk indicates a nonspecific band.

To determine the mechanism that yeast cells utilize to regulate Cth2 protein stability, we expressed Flag_2_-Cth2 in cells lacking the drug efflux pump gene *PDR5*, which are highly sensitive to the proteasome inhibitor MG132 (13). Yeast cells were cultivated in the presence or absence of MG132 under both iron-replete and iron-depleted conditions. As previously reported, Cth2 protein levels were dramatically upregulated in response to iron scarcity (3) (Fig. 6A). The addition of the MG132 proteasome inhibitor to the iron-deficient cells elevated Cth2 protein abundance, whereas little increase was observed under iron-replete conditions (Fig. 6A). To further address the relevance of the proteasome in Cth2 protein turnover, we used a temperature-sensitive *pre1-1* strain, which is defective in an essential subunit of the proteasome when grown at 37°C. We determined Cth2 protein stability at the restrictive temperature in the *pre1-1* strain and an isogenic wild-type strain. Cth2 protein displayed a half-life of 10 min at 37°C when expressed in the control strain, whereas it was highly stabilized in the *pre1-1* strain (Fig. 6B). Taken together, these data demonstrate that Cth2 protein is degraded via the proteasome.

**FIG 7:**
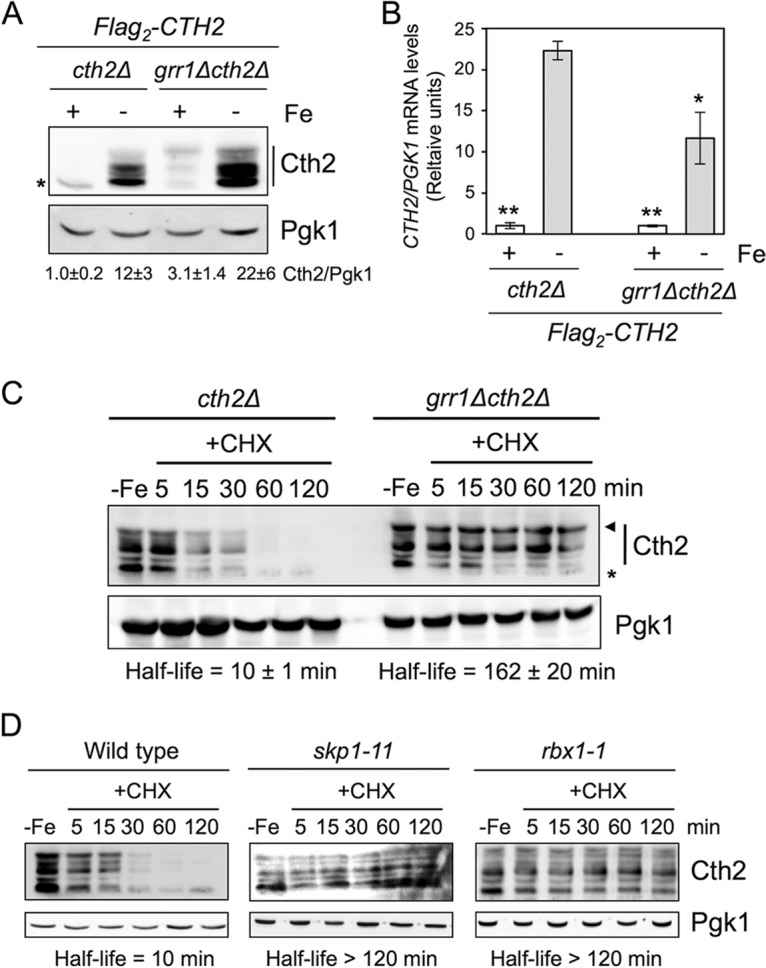
Components of the SCF^Grr1^ ubiquitin ligase complex are required for Cth2 protein degradation. (A and B) *GRR1* deletion increases Cth2 protein levels but not *CTH2* mRNA levels. Yeast *cth2*Δ (SPY25) and *grr1*Δ *cth2*Δ (SPY790) cells transformed with pRS416-Flag_2_-Cth2 plasmid were grown in SC-ura (+Fe) or SC-ura with 100 μM BPS (−Fe). (A) Proteins were extracted and analyzed as described in the legend of Fig. 1C, and the average Cth2 protein half-life was determined (*n* = 3 for −Fe experiments and *n* = 2 for +Fe experiments). (B) mRNAs were extracted and analyzed as described in the legend of Fig. 3, except that *PGK1* mRNA was used as a loading control. Values are relative to the values for *cth2*Δ cells grown with Fe. The means ± standard deviations for three independent biological experiments are shown. Values that are statistically significantly different (*P* < 0.005) for cells grown in +Fe and –Fe conditions are indicated by two asterisks. Values that are significantly different (*P* = 0.054) in *cth2*Δ and *grr1*Δ *cth2*Δ cells in –Fe conditions are indicated by a single asterisk. (C) Grr1 F-box protein promotes Cth2 protein turnover. *cth2*Δ (SPY25) and *grr1*Δ *cth2*Δ (SPY790) cells transformed with pRS416-Flag_2_-Cth2 were grown in iron-deficient conditions, treated with cycloheximide (CHX), and analyzed as described in the legend of Fig. 5. The arrowhead indicates the potentially phosphorylated Cth2 protein. The asterisk indicates a nonspecific band. Cth2 protein levels were determined, and the half-life was calculated. The results from a representative experiment are shown. The average half-life was calculated for each replicate. Then the average and standard deviation were determined (*n* = 3). (D) Skp1 and Rbx1 proteins promote Cth2 protein turnover. Wild-type (Y80), *skp1-11* (Y552), and *rbx1-1* cells transformed with pRS416-Flag_2_-Cth2 were grown in iron-deficient conditions, treated with CHX, and analyzed as described in the legend of Fig. 6B. Cth2 protein levels and the half-life were determined.

A previous genome-wide screen devoted to isolate ubiquitinated substrates in complex with their E3 ubiquitin ligase identified Cth2 protein as a potential substrate for Grr1, the substrate adaptor component of the SCF^Grr1^ E3 ubiquitin ligase complex (14). To ascertain whether Grr1 regulates Cth2 protein abundance, we determined the steady-state Cth2 protein levels in *grr1*Δ cells grown under iron-sufficient and iron-deficient conditions. Again, we observed that Cth2 protein was barely detected under iron-sufficient conditions but was highly expressed upon iron depletion (Fig. 7A). Furthermore, *grr1*Δ cells displayed an increase in Cth2 protein abundance compared to *GRR1*-expressing cells grown in both iron sufficiency and starvation (Fig. 7A). Importantly, Cth2 protein upregulation in iron-deficient *grr1*Δ cells was not due to an increase in *CTH2* mRNA levels. Instead, under iron starvation conditions, *CTH2* transcript abundance diminished in *grr1*Δ cells compared to *GRR1*-expressing cells (Fig. 7B). Overall, these results were consistent with Grr1 regulating Cth2 protein abundance at a posttranslational level. Therefore, we determined Flag_2_-Cth2 protein stability in wild-type cells and cells lacking Grr1 protein. We observed that the half-life of the Cth2 protein increased from 10 min to more than 2 h when the *GRR1* gene was deleted (Fig. 7C), demonstrating that the Grr1 F-box protein is a major factor that determines Cth2 protein stability under iron-deficient conditions.

The Grr1 F-box protein is the substrate recognition module of the SCF^Grr1^ ubiquitin ligase complex, which is composed of the cullin scaffold Cdc53 that bridges the adaptor protein Skp1 and the RING finger protein Rbx1 (15, 16). Thus, we hypothesized that cells defective in any of the SCF^Grr1^ components would increase Cth2 protein stability. To test this, we used the *skp1-11* and *rbx1-1* yeast strains, which express temperature-sensitive alleles of the essential genes *SKP1* and *RBX1*. We observed that the stability of Cth2 protein dramatically increased in *skp1-11* and *rbx1-1* mutants at the restrictive temperature (Fig. 7D). Taken together, these results demonstrate that the SCF^Grr1^ E3 ubiquitin ligase promotes the degradation of Cth2 protein during iron deficiency.

### The Grr1 F-box protein is necessary for optimal growth under iron-deficient and respiratory conditions.

**FIG 8:**
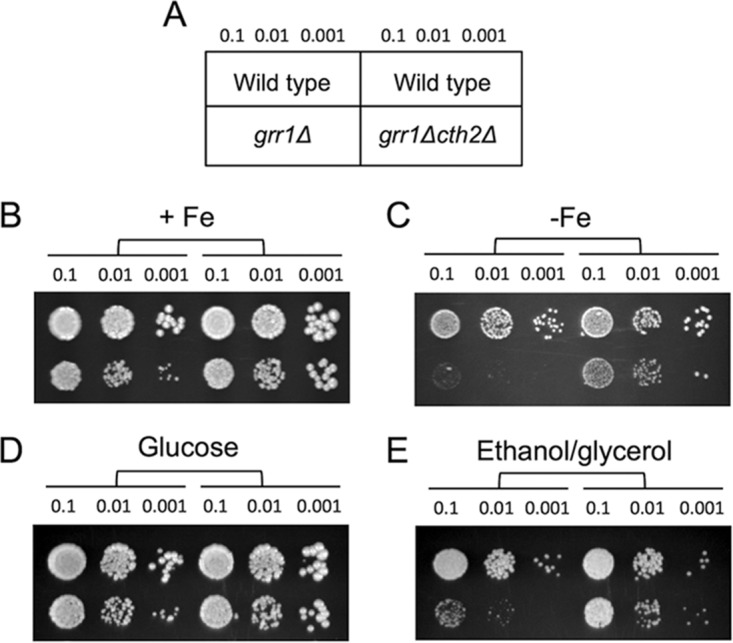
The *GRR1* requirement in low-iron and respiratory conditions is rescued by *CTH2* deletion. Wild-type (BY4741), *grr1*Δ (SPY757), and *grr1*Δ *cth2*Δ (SPY790) cells (A) were grown in liquid SC to exponential phase and spotted in 10-fold serial dilutions on SC (+Fe) (B), SC containing 1 mM Ferrozine (−Fe) (C), yeast extract-peptone-dextrose (YPD) (Glucose) (D), and yeast extract-peptone-ethanol-glycerol (YPEG) (Ethanol/glycerol) (E). The numbers (0.1, 0.01, 0.001) indicate the OD of the liquid drop spotted on the plate. Two biological replicates were incubated at 30°C for 3 days (6 days in the case of Ferrozine) and then photographed.

We have shown here that, similarly to Cth2 serine mutants, *grr1*Δ cells accumulate Cth2 protein (Fig. 1 and Fig. 7). Given that Cth2 serine mutants display a growth defect under low-iron conditions due to elevated levels of functional Cth2 protein (Fig. 2 and Fig. 5), we decided to assay the growth of *grr1*Δ cells under iron-deficient conditions (Fig. 8A). Remarkably, we observed a dramatic growth defect for *grr1*Δ cells when iron bioavailability was limited (Fig. 8B and C). In agreement with the elevated levels of functional Cth2 being the reason for this growth defect, deletion of *CTH2* gene rescued *grr1*Δ growth in low-iron media (Fig. 8C). This rescue was only partial because yeast cells solely lacking *CTH2* display a growth defect under iron-depleted conditions (3) (Fig. 8C). Among Cth2 target mRNAs, genes within the mitochondrial electron transport chain are considered preferential substrates (3). Therefore, we considered the possibility that, in response to iron deficiency, Cth2 would promote the repression of mitochondrial respiration, which consumes elevated amounts of iron but is dispensable for yeast cell growth. Thus, we tested the growth of *grr1*Δ cells under respiratory conditions by using a medium containing the nonfermentable ethanol and glycerol carbon sources instead of glucose. Yeast strains without *GRR1* exhibited a growth defect under respiring (ethanol and glycerol [ethanol/glycerol]) conditions compared to fermentative (glucose) conditions (Fig. 8D and E). Again, *CTH2* deletion partially rescued growth in ethanol/glycerol probably due to a release in the repression of respiration. All together, these results demonstrate that Grr1 protein plays a relevant function in the adaptation of yeast cells to iron deficiency and respiratory conditions by limiting Cth2 protein levels.

### The Grr1 ubiquitin ligase preferentially interacts with phosphorylated Cth2.

**FIG 9:**
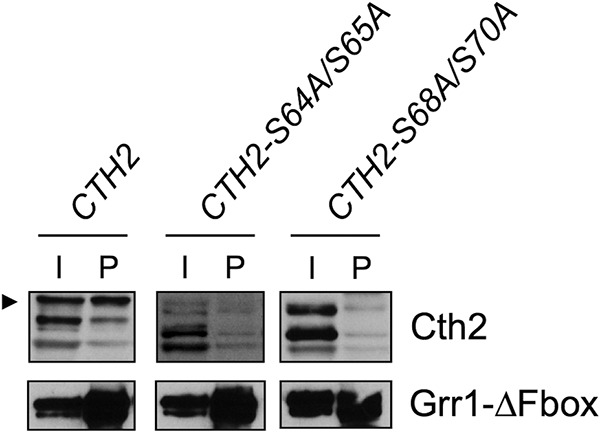
Cth2 serine residues S64/S65 and S68/S70 are required for the interaction with Grr1 protein. *grr1*Δ *cth2*Δ (SPY790) cells carrying pYES-Grr1ΔFbox-Flag plasmid were transformed with plasmid pRS413-Myc_2_-Cth2, pRS413-Myc_2_-Cth2-S64A/S65A, or pRS413-Myc_2_-Cth2-S68A/S70A. Yeast transformants were first grown in SC medium containing raffinose, but without uracil and histidine (SCRaf-ura-his) with 100 μM BPS for 3 h. Then, galactose was added to a final 2% (wt/vol) concentration, and incubation was continued for 3 additional hours. The cells were collected, and proteins were extracted and quantified. Flag-Grr1ΔFbox protein was collected with anti-Flag M2 magnetic beads. Myc_2_-Cth2 and Flag-Grr1ΔFbox protein levels were determined by Western blotting in whole-cell extracts (input [I] and Flag-immunoprecipitated samples (precipitated [P]). The arrowhead indicates the potentially phosphorylated Cth2 protein.

Given that the Grr1 F-box protein targets only phosphorylated substrates through its leucine-rich motif (17), we ascertained whether Cth2 phosphorylation was necessary for Grr1 recruitment. For this purpose, we expressed a truncated version of Grr1 protein lacking its F-box domain (Grr1ΔFbox), which recognizes its substrates but is unable to promote their degradation due to lack of interaction with the Skp1 linker protein (14). Flag-tagged Grr1ΔFbox protein was coexpressed with Myc-tagged Cth2, Cth2-S64A/S65A, or Cth2-S68A/S70A proteins, and Cth2 protein levels were determined by immunoblotting before (I lanes) and after (P lanes) Grr1 immunoprecipitation (Fig. 9). Since these cells lacked a fully functional Grr1 protein, they preferentially accumulated slow-migrating Cth2 forms that may correspond to phosphorylated Cth2 (Fig. 9, input [I] lanes). More important, Grr1ΔFbox pulldown recruited wild-type Cth2, mostly in its phosphorylated form, whereas the interaction was considerably reduced when the Cth2 S64/S65 or S68/S70 residues were mutagenized to alanine (Fig. 9, immunoprecipitated [P] lanes). These results strongly suggest that Grr1 specifically recognizes phosphorylated Cth2 and promotes its degradation by the proteasome.

### The casein kinase Hrr25 phosphorylates and promotes Cth2 turnover.

**FIG 10:**
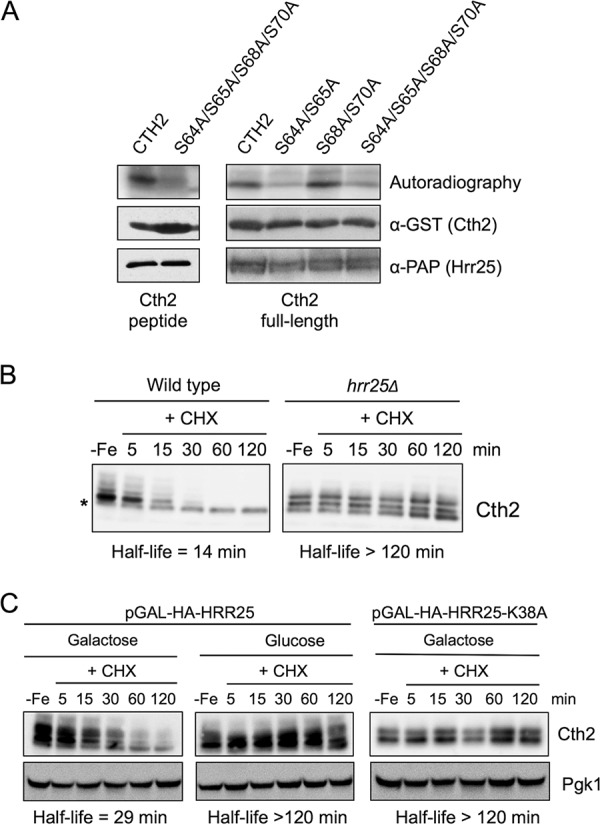
The Hrr25 casein kinase phosphorylates Cth2 and promotes its degradation. (A) Hrr25 phosphorylates Cth2 *in vitro*. Purified GST-Cth2 and GST-Cth2-S64A/S65A/S68A/S70A peptides (left) and purified GST-Cth2, GST-Cth2-S64A/S65A, GST-Cth2-S68A/S70A, and GST-Cth2-S64A/S65A/S68A/S70A proteins (right) were incubated with Hrr25-TAP in the presence of [γ-^32^P]ATP. Phosphorylated proteins and peptides were resolved by SDS-PAGE and detected by autoradiography. α-GST, anti-GST antibody; PAP, peroxidase anti-peroxidase. (B) Hrr25 enhances Cth2 protein degradation *in vivo*. Wild-type (AH326) and *hrr25*Δ (SPY892) cells transformed with the pRS416-Flag_2_-Cth2 plasmid were grown in SC-ura with 100 μM BPS. (C) Hrr25 kinase activity is required for Cth2 protein degradation *in vivo*. Yeast *hrr25*Δ (SPY892) cells transformed with either pGAL1-3HA-HRR25 or pGAL1-3HA-HRR25-K38A plasmid were grown overnight in SCRaf-ura-leu, and then cells were transferred to either SC-ura-leu (Glucose) or SC medium with galactose, and without uracil and leucine (SCGal-ura-leu) (Galactose) with 100 μM BPS to exponential phase. For panels B and C, CHX was added to stop protein translation, and Cth2 protein levels were extracted and analyzed as described in the legend of Fig. 1C. The average half-life was calculated (*n* = 2), and a representative immunoblot is shown.

To identify potential kinases that phosphorylate Cth2 protein to promote its degradation by the proteasome, we performed a systematic phosphorylation screen. Specifically, we purified a collection of 123 tandem affinity purification (TAP)-tagged protein kinases from yeast cells grown under iron-deficient conditions, and then we assayed *in vitro* their ability to phosphorylate Cth2 and Cth2-S64A/S65A/S68A/S70A mutant peptides encompassing Glu-62 to Asn-74 (Fig. 10A, Cth2 peptide). We observed that the casein kinase I (CKI) Hrr25 phosphorylated the wild-type Cth2 peptide, whereas the Cth2-S64A/S65A/S68A/S70A mutant peptide was phosphorylated to a lesser extent (Fig. 10A, Cth2 peptide). To further characterize the Cth2 residues phosphorylated by Hrr25, we performed an *in vitro* kinase assay with full-length glutathione *S*-transferase (GST)-Cth2, GST-Cth2-S64A/S65A, GST-Cth2-S68A/S70A, and GST-Cth2-S64A/S65A/S68A/S70A fusion proteins purified from Escherichia coli. We observed that Hrr25 phosphorylated Cth2 and Cth2-S68A/S70A proteins, whereas the Cth2-S64A/S65A/S68A/S70A and Cth2-S64A/S65A mutant proteins displayed a strong reduction in phosphorylation (Fig. 10A). This result pointed to Cth2 S64 and S65 as preferential Hrr25 target residues over S68 and S70.

These results also suggested that Hrr25 kinase could influence Cth2 protein stability *in vivo*. To test this possibility, we grew wild-type and *hrr25*Δ mutant cells under iron-depleted conditions and performed Cth2 protein stability assays. We observed that the deletion of the *HRR25* gene augmented Cth2 protein half-life from 12 min to more than 2 h (Fig. 10B). To further address Cth2 regulation by Hrr25, we expressed wild-type *HRR25* and its catalytically inactive *HRR25-K38A* allele under the control of the *GAL1* promoter under iron-depleted conditions and determined Cth2 stability (18). In agreement with our previous observations, the expression of *HRR25* promoted the degradation of Cth2 (Fig. 10C, *HRR25* cells in galactose-containing medium), whereas Cth2 protein half-life was more than 2 h in the absence of *HRR25* expression (Fig. 10C, *HRR25* cells in glucose-containing medium). Importantly, the expression of the *HRR25-K38A* kinase-dead allele did not enhance Cth2 protein turnover in galactose-containing medium (Fig. 10C, *HRR25-K38A*). These results demonstrate that Cth2 phosphorylation by the casein kinase Hrr25 is essential to promote Cth2 protein degradation.

## DISCUSSION

The adaptation of eukaryotic cells to iron deficiency requires sophisticated regulatory mechanisms. In S. cerevisiae, the posttranscriptional factor Cth2 is tightly regulated to allow the appropriate utilization of the iron cofactor in specific metabolic pathways (3, 11, 12). Cth2 is expressed at very low levels in iron-replete conditions, whereas the Aft1 and Aft2 transcription factors strongly activate *CTH2* expression upon iron limitation (3, 19). However, the iron-deregulated *CTH2* overexpression achieved with a constitutive or galactose-inducible promoter causes toxicity (5, 10). Therefore, *CTH2* expression is limited by a negative-feedback regulation in which Cth2 protein binds to AU-rich elements (AREs) within its own transcript and promotes its degradation (12). In the absence of this autoregulatory mechanism, yeast cells increase Cth2 protein abundance and diminish fitness in iron-limiting conditions (12). Here, we have shown that Cth2 expression is also controlled through its specific phosphorylation and degradation by the proteasome. When Cth2 serine residues are mutagenized or a component of the E3 ubiquitin ligase complex SCF^Grr1^ that recognizes phospho-Cth2 is absent, Cth2 protein stability increases, leading to strong growth defects in iron-deficient cells. Therefore, the strict control of Cth2 expression levels is crucial during iron deficiency.

The molecular reasons underlying Cth2 overexpression toxicity have not been fully elucidated. This work and previous studies have shown that mutagenesis or deletion of Cth2 tandem zinc fingers (TZFs), which mediate specific binding to AREs within the target mRNAs, rescues the slow growth phenotype of Cth2-overexpressing cells (5, 10). These results suggest that the increased levels of a functional Cth2 protein could be accelerating the degradation of target mRNAs. We do not observe an enhanced turnover of Cth2 targets, including *SDH4*, *HEM15*, and the essential transcripts *RNR2* and *RLI1* (Fig. 3). However, we cannot dismiss the possibility that changes in mRNA levels of other transcripts are responsible for detrimental growth. The nucleocytoplasmic shuttling of Cth2 protein is required to promote targeted mRNA turnover (11). However, Cth2 does not contain any nuclear export signal and relies on its cotranscriptional binding to ARE-containing mRNAs to exit the nucleus, and consequently, Cth2 TZF mutants are trapped in the nucleus (11). These observations raise the possibility that the overexpression of Cth2 could be titrating components of the mRNA degradation machinery during its nucleocytoplasmic shuttling, whereas the disruption of its TZF would trap Cth2 in the nucleus and rescue cellular viability. Deciphering strategies that separate shuttling from mRNA degradation would help to differentiate between these two possibilities.

Here we demonstrate that the SCF^Grr1^ E3 ubiquitin ligase complex plays a key function in yeast iron homeostasis by recognizing phosphorylated Cth2 protein and targeting it for degradation by the proteasome. The physiological relevance of SCF^Grr1^ regulation is corroborated by the severe *grr1*Δ growth defect under iron-deficient conditions, which is rescued by *CTH2* deletion (Fig. 8). In addition to Cth2, a recent global study has also identified Dre2, a component of the cytosolic iron-sulfur protein assembly machinery, as a Grr1 target (14). The implication of E3 ubiquitin ligases in iron metabolism extends to mammals, where the SCF^FBXL5^ complex promotes the proteasomal degradation of the iron regulatory proteins IRP2 and apo-IRP1 (20, 21), and higher plants, where the RING-type BRUTUS and HRZ proteins play central roles in the iron homeostasis of *Arabidopsis* and rice, respectively (22, 23). Collectively, these data reinforce the notion that E3 ubiquitin ligase complexes occupy a privileged position in the regulation of eukaryotic iron homeostasis.

The shuttling of Cth2 protein is essential for targeted mRNA degradation (11). Cth2 enters the nucleus in order to cotranscriptionally associate with the mRNAs that will be specifically degraded later (11). Regarding subcellular distribution, we have previously reported that Cth2 proteins lacking their 89 amino-terminal amino acids still localize to both the nucleus and cytoplasm (11), which suggests that the mutagenesis of the S64/S65/S68/S70 residues does not alter Cth2 nucleocytoplasmic shuttling. Consistent with this, we show here that the phosphorylation of yeast Cth2 protein at a patch of serine residues (S64/S65/S68/S70) does not alter targeted mRNA turnover but Cth2 protein stability. Interestingly, the Cth2-C190R mutant protein, which cannot bind mRNAs and is trapped in the nucleus, is still phosphorylated (12). These results suggest that phosphorylation occurs before Cth2 associates with its mRNA targets. Furthermore, we previously observed that Cth2 protein accumulated in its phosphorylated form when forced to localize to the cytoplasm, whereas it was lost when Cth2 import into the nucleus was enhanced (11). Altogether, these data suggest that Cth2 is synthesized in the cytoplasm and that at least a fraction of it is phosphorylated even before its entrance into the nucleus. These results reinforce phosphorylation as a mechanism to control TZF-type proteins in different ways. Although much progress has been made, additional approaches are necessary to completely understand the regulatory role and the impact that phosphorylation exerts on TZF-containing proteins, particularly in an iron-deficient situation.

By using both *in vitro* and *in vivo* approaches, we have revealed that Cth2 is phosphorylated by the conserved casein kinase Hrr25, which is involved in a wide range of cellular functions, including ribosome maturation, vesicle trafficking and endocytosis, autophagy, DNA repair, and chromosome segregation during meiosis (24–26). We have observed that Cth2 protein is highly stable in *hrr25*Δ mutant cells, although its electrophoretic mobility is not altered after the addition of cycloheximide (Fig. 10B and C). These results suggest that Cth2 protein is still phosphorylated in *hrr25*Δ cells, implicating other kinases in Cth2 phosphorylation. Consistent with this, the separate mutagenesis of Cth2 S64/S65 and S68/S70 residues has uncovered that Hrr25 is responsible for the phosphorylation of only the S64 and S65 sites, whereas the other pair of serine residues seems more promiscuous (Fig. 10). Further observations highlight the interplay existing between the two pairs of serine residues. In particular, the stabilization observed for the Cth2 quadruple serine mutant was not as pronounced as the one displayed for the Cth2 double mutants (Fig. 5; see also Fig. S2D in the supplemental material). Consistent with this, the Cth2-S64A/S65A/S68A/S70A steady-state protein levels increased only 1.5-fold, and the growth defect was not as severe as the growth defect observed for the cells expressing the Cth2-S64A/S65A and Cth2-S68A/S70A double mutants (Fig. S2). In any case, these results indicate that the destabilization of Cth2 protein requires the simultaneous phosphorylation at both serine pairs by different kinases. Although we still do not understand its physiological significance, we could speculate that this dual targeting would allow the stabilization of Cth2 protein in an active state in response to certain situations by altering only one of these phosphorylation events. The further characterization of phosphorylation events within TZF-containing proteins would help to understand how these regulatory factors are posttranslationally controlled.

## MATERIALS AND METHODS

### Yeast strains and growth conditions.

The Saccharomyces cerevisiae strains used in this work are listed in Table S1 in the supplemental material. To delete *CTH2* and *HRR25* genes in particular strains, we generated *cth2*::*hphB*, *cth2*::*HisMX6*, and *hrr25*::*KanMX4* cassettes as previously described (27, 28). Yeast precultures were grown to exponential phase at 30°C in synthetic complete medium (SC) lacking specific requirements. To regulate iron availability, the cells were incubated in SC (with Fe) or SC supplemented with 100 μM concentration of the Fe^2+^-specific chelator bathophenanthroline disulfonic acid disodium or bathophenanthroline disulfonic acid (BPS) (Merck) (without Fe). To perform growth assays in solid media, yeasts were spotted at an optical density at 600 nm (OD_600_) of 0.1 and after 1:10 and 1:100 dilutions in SC containing 0.7 to 1.1 mM concentrations of the Fe^2+^-specific chelator Ferrozine (Merck).

10.1128/mBio.01694-18.4 TABLE S1 Yeast strains used in this study. Download Table S1, DOCX file, 0.02 MB. Copyright © 2018 Romero et al. 2018 Romero et al. This content is distributed under the terms of the Creative Commons Attribution 4.0 International license.

### Plasmids.

The plasmids used in this work are listed in Table S2. Flag-tagged *CTH2* was amplified from pSP413 plasmid and cloned into pRS426 to obtain pSP421 plasmid. Two copies of the Myc epitope were introduced into a NotI restriction site after the *CTH2* start codon to generate a Myc_2_-tagged Cth2 allele, which was cloned into pRS413 to obtain pSP886 plasmid. Plasmids pSP413 and pSP886 were used as the templates to mutagenize Cth2 residues S64, S65, S68, S70, and C190 by using either the overlapping extension method (3) or the Phusion site-directed mutagenesis kit (Thermo Fisher Scientific). In all cases, BamHI and XhoI restriction sites were used to clone *CTH2* amplified sequences. Other plasmids used in this work have been described previously (3, 4, 12, 14, 29) (Table S2). All PCR amplifications were performed with the Phusion polymerase (Thermo Fisher Scientific).

### Mass spectrometry.

Immunoprecipitates were precipitated by the addition of ice-cold acetone and digested by the sequential addition of lys-C and trypsin proteases as previously described (30). Peptide digests were then analyzed by liquid chromatography coupled to tandem mass spectrometry (LC-MS/MS) on an LTQ-Orbitrap XL instrument (Thermo Fisher Scientific) (30). Phosphorylation sites were identified with the ProLuCID database search algorithm using a differential modification search that considered a mass shift of 79.9663 on serines, threonines, and tyrosines (31). Phosphosite validation and localization were performed using Debunker and Ascore, respectively (32, 33).

10.1128/mBio.01694-18.5 TABLE S2 Plasmids used in this study. Download Table S2, DOCX file, 0.1 MB. Copyright © 2018 Romero et al. 2018 Romero et al. This content is distributed under the terms of the Creative Commons Attribution 4.0 International license.

### Western blotting.

Total protein extracts were obtained by using the alkali method (34). Equivalent amounts of protein were resolved on SDS-polyacrylamide gels and transferred to nitrocellulose membranes. Ponceau S staining was used to assess protein transfer and loading. The Flag epitope was detected using a horseradish peroxidase (HRP)-conjugated antibody. The primary antibodies used in this study included anti-Flag (Merck), anti-c-*myc* (Roche Applied Science), anti-Pgk1 (Thermo Fisher Scientific), anti-glutathione *S*-transferase (anti-GST) (Merck), and anti-peroxidase-antiperoxidase (anti-PAP) (Merck). Immunoblots were developed with horseradish peroxidase (HRP)-labeled secondary antibodies and the ECL Select Western blotting detection kit (GE Healthcare Life Sciences). Images were scanned with an ImageQuant LAS 4000 mini biomolecular imager (GE Healthcare Life Sciences), and specific signals were quantified and processed with ImageQuant TL analysis software (GE Healthcare Life Sciences).

### Coimmunoprecipitation.

To study the interaction between Cth2 and Grr1 proteins by coimmunoprecipitation (CoIP), *grr1*Δ *cth2*Δ cells were cotransformed with pYES-Grr1ΔFbox-Flag plasmid and pRS413-Myc_2_-Cth2, pRS413-Myc_2_-Cth2-S64A/S65A, or pRS413-Myc_2_-Cth2-S68A/S70A plasmid. The cells were grown in synthetic complete medium lacking uracil and histidine (SC-ura-his) without glucose but containing 100 µM BPS and 2% raffinose for 3 h. Then 2% galactose was added, and the cultures were incubated 3 additional hours. The cells were harvested and resuspended in 700 μl HEPES lysis buffer (25 mM HEPES [pH 7.5], 150 mM NaCl, 1 mM EDTA, 17 μg/ml phenylmethylsulfonyl fluoride [PMSF], 5 mM sodium fluoride, 80 mM β-glycerophosphate, 1 mM sodium orthovanadate, and a Complete protease inhibitor tablet [Thermo Fisher Scientific]). The cells were lysed by bead beating in a cold block for six cycles with one cycle consisting of 1 min of bead beating and 2 min on ice, and the cells were cleared by centrifugation at 4°C. Protein concentrations were determined by the Bradford method, and equal amounts of proteins were incubated with 30-μl slurry of anti-Flag M2 magnetic beads (Merck) while rotating at 4°C overnight. Magnetic beads were collected on a magnetic rack and washed three times with 700 μl of 1× phosphate-buffered saline (PBS) buffer containing 0.1% Nonidet P-40 (NP-40). Then, proteins were eluted by mild vortexing in 1× PBS buffer containing 500 ng/ml 3×Flag peptide (Merck) for 30 min. Total protein extract and elution extract were analyzed by Western blotting.

### Protein purification and *in vitro* kinase assays.

Short Cth2 peptides were designed to contain the wild-type (WT) amino acids S64, S65, S68, and S70 or the corresponding alanine mutant (Ser to Ala [S/A]). The sequence NH_2_-EI[S/A][S/A]AV[S/A]F[S/A]PPKN-COOH contains Cth2 amino acid residues from 62 to 74. Oligonucleotides with the corresponding WT or S/A peptide sequences were cloned into pGEX6-P1 vector to obtain recombinant GST-fused peptides. Recombinant GST-Cth2-fused peptides and proteins (GST-Cth2-WT and GST-Cth2-S64A/S65A/S68A/S70A) were expressed in Escherichia coli BL21 and purified using glutathione-Sepharose beads (GE Healthcare Life Sciences) in STET buffer (10 mM Tris [pH 8.0], 100 mM NaCl, 1 mM EDTA [pH 8.0], 5% Triton X-100, 2 mM dithiothreitol [DTT], 1 mM phenylmethylsulfonyl fluoride, 1 mM benzamidine, 2 μg/ml leupeptin, 2 μg/ml pepstatin). Bound proteins were then eluted in 10 mM reduced glutathione, 50 mM Tris-HCl (pH 9.5), and 2 mM DTT.

Kinase-tagged strains from the yeast tandem affinity purification (TAP) collection (35) were grown to mid-log phase for 6.5 h at 30°C in 50 ml of yeast extract-peptone-dextrose (YPD) treated with 100 μM BPS. Cells were collected by brief centrifugation at 4°C. Proteins were extracted in buffer A (50 mM Tris-HCl [pH 7.5], 150 mM NaCl, 15 mM EDTA, 15 mM EGTA, 2 mM DTT, 0.1% Triton X-100, 1 mM PMSF, 1 mM benzamidine, 2 μg/ml leupeptin, 2 μg/ml pepstatin) and incubated with rabbit IgG-agarose beads (Merck). After the beads were washed, kinase-bound beads were used for the *in vitro* kinase assay. Purified TAP-tagged kinase was preactivated with kinase buffer (50 mM Tris-HCl [pH 7.5], 10 mM MgCl_2_, 2 mM DTT, and 50 µM ATP) for 5 min at 30°C. One microgram of the substrate GST-Cth2 full-length proteins or short peptides was added to the kinase-bound bead mixture together with radiolabeled [γ-^32^P]ATP (0.1 µCi/µl) and incubated for 30 min at 30°C. The reaction was stopped by the addition of 5× SDS loading buffer. Labeled proteins were resolved by SDS-PAGE, transferred to a nylon membrane, and detected by autoradiography. GST-fused proteins and TAP-tagged kinases were detected by Western blotting.

### Other protein analyses.

To determine Cth2 protein stability, yeast cells were grown in iron-replete or iron-deficient conditions for 6 h. Then, protein translation was stopped by the addition of cycloheximide (CHX) to a final concentration of 50 µg/ml. Cells were harvested at the indicated time points and processed for Western blotting. For proteasome inhibition, *pdr5*Δ cells transformed with pRS416-Flag_2_-CTH2 plasmid were treated with the proteasomal inhibitor MG132 (Merck) at a final concentration of 100 µM or with dimethyl sulfoxide (DMSO) as a negative control for 1 h. For treatments with λ phosphatase, total proteins of yeast cells expressing Flag_2_-Cth2 (Cth2 tagged with two copies of the Flag epitope) or Flag_2_-Cth1 were extracted from cell pellets using a Tehtnica Millmix 20 bead beater (Domel) in lysis buffer (50 mM HEPES, 150 mM KCl, 1 mM EDTA pH 8.0, 10% glycerol, 0.1% NP-40) and 0.3 ml of glass beads. Protein extract (30 μg) was treated with λ phosphatase (New England Biolabs) according to the manufacturer’s recommendations in the presence or absence of protein inhibitors (66 mM NaF and 66 mM EDTA). In all cases, Cth2 protein levels were determined by Western blotting. Clustal Omega (European Bioinformatics Institute) was used to align Cth1 and Cth2 proteins.

### RNA analyses.

Total RNA extraction and cellular mRNA levels were determined by reverse transcription-quantitative real-time PCR (RT-qPCR) as previously described (36). The primers used for RT-qPCR are listed in Table S3. The data and error bars represent the averages and standard deviations for three independent biological samples.

10.1128/mBio.01694-18.6 TABLE S3 Oligonucleotides used for RT-qPCR in this study. Download Table S3, DOCX file, 0.01 MB. Copyright © 2018 Romero et al. 2018 Romero et al. This content is distributed under the terms of the Creative Commons Attribution 4.0 International license.

### Statistical analyses.

Each sample analyzed contained approximately 10^8^ yeast cells. The number of independent biological replicates is indicated in each figure legend. Two-tailed Student *t* tests were applied to evaluate statistical significance. The asterisks indicate statistically significant differences with the specified *P* value.

## References

[1] Baynes RD, Bothwell TH 1990 Iron deficiency. Annu Rev Nutr 10:133–148. doi:10.1146/annurev.nu.10.070190.001025. 2200460

[2] Muckenthaler MU, Rivella S, Hentze MW, Galy B 2017 A red carpet for iron metabolism. Cell 168:344–361. doi:10.1016/j.cell.2016.12.034. 28129536 PMC5706455

[3] Puig S, Askeland E, Thiele DJ 2005 Coordinated remodeling of cellular metabolism during iron deficiency through targeted mRNA degradation. Cell 120:99–110. doi:10.1016/j.cell.2004.11.032. 15652485

[4] Puig S, Vergara SV, Thiele DJ 2008 Cooperation of two mRNA-binding proteins drives metabolic adaptation to iron deficiency. Cell Metab 7:555–564. doi:10.1016/j.cmet.2008.04.010. 18522836 PMC2459314

[5] Thompson MJ, Lai WS, Taylor GA, Blackshear PJ 1996 Cloning and characterization of two yeast genes encoding members of the CCCH class of zinc finger proteins: zinc finger-mediated impairment of cell growth. Gene 174:225–233. doi:10.1016/0378-1119(96)00084-4. 8890739

[6] Ramos-Alonso L, Romero AM, Soler MÀ, Perea-García A, Alepuz P, Puig S, Martínez-Pastor MT 2018 Yeast Cth2 protein represses the translation of ARE-containing mRNAs in response to iron deficiency. PLoS Genet 14:e1007476. doi:10.1371/journal.pgen.1007476. 29912874 PMC6023232

[7] Brooks SA, Blackshear PJ 2013 Tristetraprolin (TTP): interactions with mRNA and proteins, and current thoughts on mechanisms of action. Biochim Biophys Acta 1829:666–679. doi:10.1016/j.bbagrm.2013.02.003. 23428348 PMC3752887

[8] Wells ML, Perera L, Blackshear PJ 2017 An ancient family of RNA-binding proteins: still important! Trends Biochem Sci 42:285–296. doi:10.1016/j.tibs.2016.12.003. 28096055 PMC5376222

[9] Sanvisens N, Bano MC, Huang M, Puig S 2011 Regulation of ribonucleotide reductase in response to iron deficiency. Mol Cell 44:759–769. doi:10.1016/j.molcel.2011.09.021. 22152479 PMC3240860

[10] Pedro-Segura E, Vergara SV, Rodriguez-Navarro S, Parker R, Thiele DJ, Puig S 2008 The Cth2 ARE-binding protein recruits the Dhh1 helicase to promote the decay of succinate dehydrogenase *SDH4* mRNA in response to iron deficiency. J Biol Chem 283:28527–28535. doi:10.1074/jbc.M804910200. 18715869 PMC2568921

[11] Vergara SV, Puig S, Thiele DJ 2011 Early recruitment of AU-rich element-containing mRNAs determines their cytosolic fate during iron deficiency. Mol Cell Biol 31:417–429. doi:10.1128/MCB.00754-10. 21135132 PMC3028617

[12] Martinez-Pastor M, Vergara SV, Puig S, Thiele DJ 2013 Negative feedback regulation of the yeast Cth1 and Cth2 mRNA binding proteins is required for adaptation to iron deficiency and iron supplementation. Mol Cell Biol 33:2178–2187. doi:10.1128/MCB.01458-12. 23530061 PMC3648069

[13] Collins GA, Gomez TA, Deshaies RJ, Tansey WP 2010 Combined chemical and genetic approach to inhibit proteolysis by the proteasome. Yeast 27:965–974. doi:10.1002/yea.1805. 20625982 PMC3566228

[14] Mark KG, Simonetta M, Maiolica A, Seller CA, Toczyski DP 2014 Ubiquitin ligase trapping identifies an SCF(Saf1) pathway targeting unprocessed vacuolar/lysosomal proteins. Mol Cell 53:148–161. doi:10.1016/j.molcel.2013.12.003. 24389104 PMC4032118

[15] Willems AR, Schwab M, Tyers M 2004 A hitchhiker’s guide to the cullin ubiquitin ligases: SCF and its kin. Biochim Biophys Acta 1695:133–170. doi:10.1016/j.bbamcr.2004.09.027. 15571813

[16] Finley D, Ulrich HD, Sommer T, Kaiser P 2012 The ubiquitin-proteasome system of Saccharomyces cerevisiae. Genetics 192:319–360. doi:10.1534/genetics.112.140467. 23028185 PMC3454868

[17] Hsiung YG, Chang HC, Pellequer JL, La Valle R, Lanker S, Wittenberg C 2001 F-box protein Grr1 interacts with phosphorylated targets via the cationic surface of its leucine-rich repeat. Mol Cell Biol 21:2506–2520. doi:10.1128/MCB.21.7.2506-2520.2001. 11259599 PMC86883

[18] Kafadar KA, Zhu H, Snyder M, Cyert MS 2003 Negative regulation of calcineurin signaling by Hrr25p, a yeast homolog of casein kinase I. Genes Dev 17:2698–2708. doi:10.1101/gad.1140603. 14597664 PMC280619

[19] Shakoury-Elizeh M, Tiedeman J, Rashford J, Ferea T, Demeter J, Garcia E, Rolfes R, Brown PO, Botstein D, Philpott CC 2004 Transcriptional remodeling in response to iron deprivation in Saccharomyces cerevisiae. Mol Biol Cell 15:1233–1243. doi:10.1091/mbc.e03-09-0642. 14668481 PMC363115

[20] Vashisht AA, Zumbrennen KB, Huang X, Powers DN, Durazo A, Sun D, Bhaskaran N, Persson A, Uhlen M, Sangfelt O, Spruck C, Leibold EA, Wohlschlegel JA 2009 Control of iron homeostasis by an iron-regulated ubiquitin ligase. Science 326:718–721. doi:10.1126/science.1176333. 19762596 PMC2929180

[21] Salahudeen AA, Thompson JW, Ruiz JC, Ma HW, Kinch LN, Li Q, Grishin NV, Bruick RK 2009 An E3 ligase possessing an iron-responsive hemerythrin domain is a regulator of iron homeostasis. Science 326:722–726. doi:10.1126/science.1176326. 19762597 PMC3582197

[22] Kobayashi T, Nagasaka S, Senoura T, Itai RN, Nakanishi H, Nishizawa NK 2013 Iron-binding haemerythrin RING ubiquitin ligases regulate plant iron responses and accumulation. Nat Commun 4:2792. doi:10.1038/ncomms3792. 24253678 PMC3905729

[23] Long TA, Tsukagoshi H, Busch W, Lahner B, Salt DE, Benfey PN 2010 The bHLH transcription factor POPEYE regulates response to iron deficiency in Arabidopsis roots. Plant Cell 22:2219–2236. doi:10.1105/tpc.110.074096. 20675571 PMC2929094

[24] DeMaggio AJ, Lindberg RA, Hunter T, Hoekstra MF 1992 The budding yeast HRR25 gene product is a casein kinase I isoform. Proc Natl Acad Sci U S A 89:7008–7012. doi:10.1073/pnas.89.15.7008. 1495994 PMC49634

[25] Schafer T, Maco B, Petfalski E, Tollervey D, Bottcher B, Aebi U, Hurt E 2006 Hrr25-dependent phosphorylation state regulates organization of the pre-40S subunit. Nature 441:651–655. doi:10.1038/nature04840. 16738661

[26] Ye Q, Ur SN, Su TY, Corbett KD 2016 Structure of the Saccharomyces cerevisiae Hrr25:Mam1 monopolin subcomplex reveals a novel kinase regulator. EMBO J 35:2139–2151. doi:10.15252/embj.201694082. 27491543 PMC5048352

[27] Longtine MS, McKenzie A III, Demarini DJ, Shah NG, Wach A, Brachat A, Philippsen P, Pringle JR 1998 Additional modules for versatile and economical PCR-based gene deletion and modification in Saccharomyces cerevisiae. Yeast 14:953–961. doi:10.1002/(SICI)1097-0061(199807)14:10<953::AID-YEA293>3.0.CO;2-U. 9717241

[28] Goldstein AL, McCusker JH 1999 Three new dominant drug resistance cassettes for gene disruption in Saccharomyces cerevisiae. Yeast 15:1541–1553. doi:10.1002/(SICI)1097-0061(199910)15:14<1541::AID-YEA476>3.0.CO;2-K. 10514571

[29] Sikorski RS, Hieter P 1989 A system of shuttle vectors and yeast host strains designed for efficient manipulation of DNA in Saccharomyces cerevisiae. Genetics 122:19–27. 2659436 10.1093/genetics/122.1.19 PMC1203683

[30] Wohlschlegel JA 2009 Identification of SUMO-conjugated proteins and their SUMO attachment sites using proteomic mass spectrometry. Methods Mol Biol 497:33–49. doi:10.1007/978-1-59745-566-4_3. 19107409

[31] Xu T, Park SK, Venable JD, Wohlschlegel JA, Diedrich JK, Cociorva D, Lu B, Liao L, Hewel J, Han X, Wong CCL, Fonslow B, Delahunty C, Gao Y, Shah H, Yates JR III. 2015 ProLuCID: an improved SEQUEST-like algorithm with enhanced sensitivity and specificity. J Proteomics 129:16–24. doi:10.1016/j.jprot.2015.07.001. 26171723 PMC4630125

[32] Lu B, Ruse C, Xu T, Park SK, Yates J III. 2007 Automatic validation of phosphopeptide identifications from tandem mass spectra. Anal Chem 79:1301–1310. doi:10.1021/ac061334v. 17297928 PMC2527591

[33] Beausoleil SA, Villen J, Gerber SA, Rush J, Gygi SP 2006 A probability-based approach for high-throughput protein phosphorylation analysis and site localization. Nat Biotechnol 24:1285–1292. doi:10.1038/nbt1240. 16964243

[34] Kushnirov VV 2000 Rapid and reliable protein extraction from yeast. Yeast 16:857–860. doi:10.1002/1097-0061(20000630)16:9<857::AID-YEA561>3.0.CO;2-B. 10861908

[35] Ghaemmaghami S, Huh WK, Bower K, Howson RW, Belle A, Dephoure N, O’Shea EK, Weissman JS 2003 Global analysis of protein expression in yeast. Nature 425:737–741. doi:10.1038/nature02046. 14562106

[36] Sanvisens N, Romero AM, An X, Zhang C, de Llanos R, Martínez-Pastor MT, Bañó MC, Huang M, Puig S 2014 Yeast Dun1 kinase regulates ribonucleotide reductase inhibitor Sml1 in response to iron deficiency. Mol Cell Biol 34:3259–3271. doi:10.1128/MCB.00472-14. 24958100 PMC4135553

