# Supplementary material for: Phosphorylation and Proteasome Recognition of the mRNA-Binding Protein Cth2 Facilitates Yeast Adaptation to Iron Deficiency
Source: mBio. 2018 Sep 18;9(5):e01694-18. doi: 10.1128/mBio.01694-18 (PMC6143738; doi:10.1128/mBio.01694-18)
Supplement: FIG S1 [file mbo005184074sf1.pdf]

A

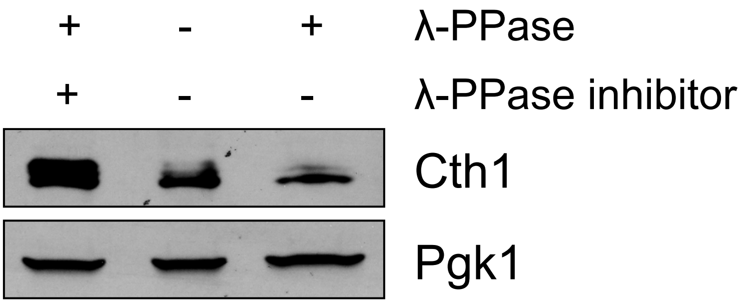

B

```
Cth2  MWAQLSYTRPESQKTDLTSLFSTDQEQNPLN---DYQYQINIRELEEYYNKTILNE--DN 55
Cth1  ---MMPNVAPNSYYLNIPNANSTSTTTSSIFSDLNKEYESKIKEIEEYYIKTLLNENTDN 57
          :. . *:*   ::..  **.   ..:    : :*: :*:*:**** **:***  **

Cth2  IQETSS--------EISSAVSFSP----PKNTNAIQPG------LLYDP------QLMNP 91
Cth1  DDSSSSEGHNINETDILSEYSPRPSPWLPSKPNCYHPLGDFKDLIISDSRPTNTLPINNP 117
       :.:**        :* *  *  *    *.:.*. :*       :: *.       : **

Cth2  FLPSAHLNSTAPTTFKKK---LEVQINPDYVPKSSQLPLTSQNLQQLSQQKPKND---AS 145
Cth1  FAGNNNISTLATTEKKRKKRSLEVEINPTYTTSAFSLPLTAENLQKLSQVDSQSTGLPYT 177
      *  . ::.: *.*  *:*   ***:*** *...: .****::***:*** ..:.     :

Cth2  FSSEKESSAQPKVKSQVQETP---KQLYKTELCESFTLKGSCPYGSKCQFAHGLGELKVK 202
Cth1  LPIQKTTKLEPCRRAPLQLPQLVNKTLYKTELCESFTIKGYCKYGNKCQFAHGLNELKFK 237
      :. :* :. :*  :: :* .    * ***********:** * **.********.***.*

Cth2  KSCKNFRTKPCVNWEKLGYCPYGRRCCFKHGDDNDIAVYVKA--GTYCNVSSTSKQSDEK 260
Cth1  KKSNNYRTKPCINWSKLGYCPYGKRCCFKHGDDKDVEIYQNANDGRSKDTALTPLPTSLA 297
      *..:*:*****:**.********:*********:*: :* :*  *   :.: *.  :.

Cth2  RSNG---RGSAKKKNLNVKVKALQRMTW 285
Cth1  PSNNDNITNLSKPRNLHTSVKALQRMTW 325
       **.    . :* :**:..*********
```

C

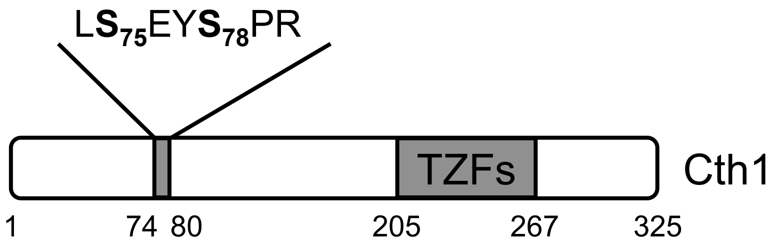

Supplemental Figure S1
