# Supplementary figures and images for: Phosphorylation and Proteasome Recognition of the mRNA-Binding Protein Cth2 Facilitates Yeast Adaptation to Iron Deficiency
Source: mBio. 2018 Sep 18;9(5):e01694-18. doi: 10.1128/mBio.01694-18 (PMC6143738; doi:10.1128/mBio.01694-18)

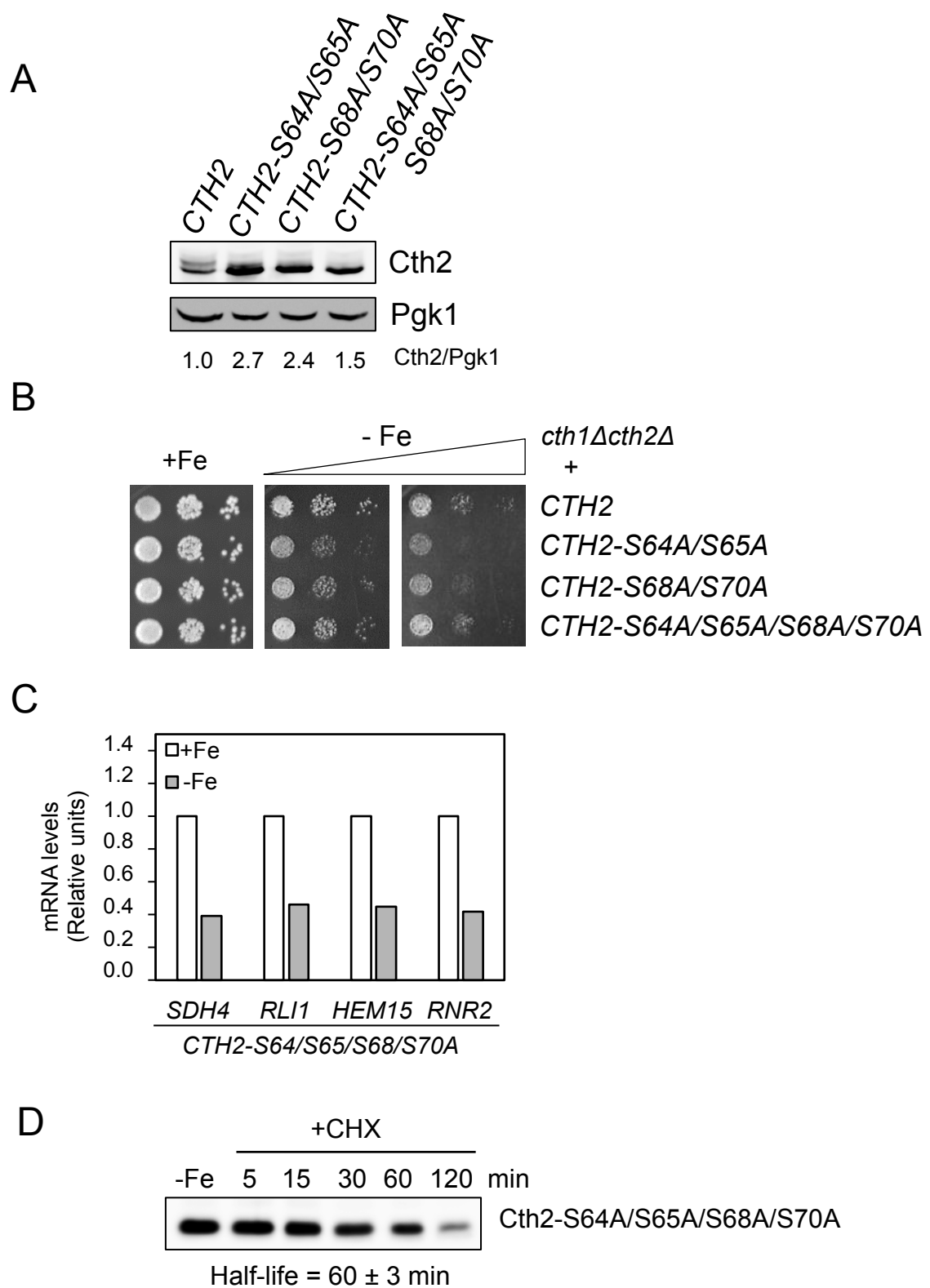

**Supplemental Figure S2**

Supplement: FIG S2 [file mbo005184074sf2.pdf]

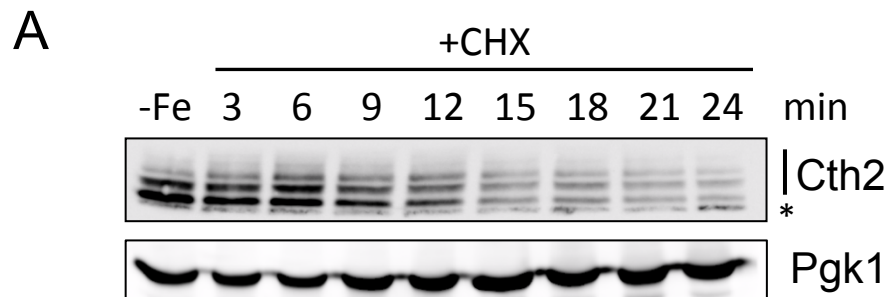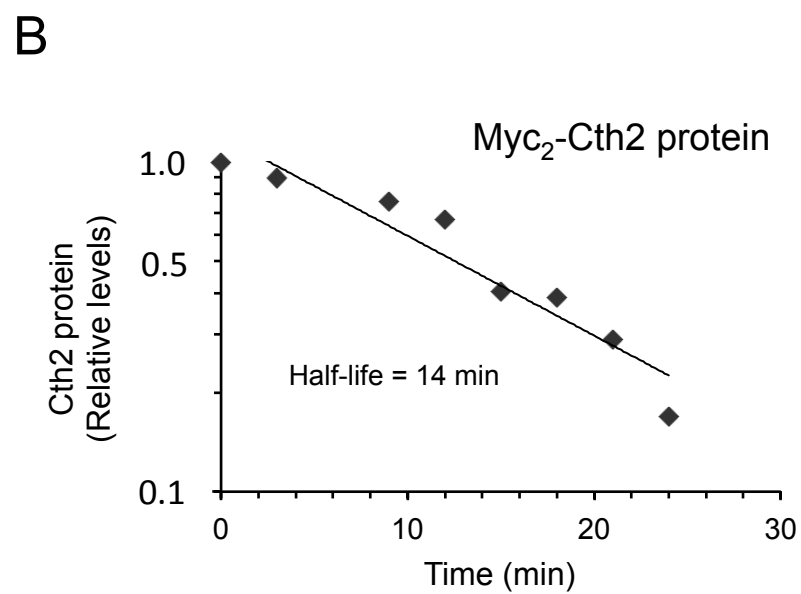

**Supplemental Figure S3**

Supplement: FIG S3 [file mbo005184074sf3.pdf]
