# Supplementary material for: Phosphorylation and Proteasome Recognition of the mRNA-Binding Protein Cth2 Facilitates Yeast Adaptation to Iron Deficiency
Source: mBio. 2018 Sep 18;9(5):e01694-18. doi: 10.1128/mBio.01694-18 (PMC6143738; doi:10.1128/mBio.01694-18)
Supplement: TABLE S1 [file mbo005184074st1.docx]

**Table S1. List of yeast strains used in this study.**

| **Strain** | **Relevant genotype** | **Source/Reference** |
| --- | --- | --- |
| BY4741 | *MATa his3Δ1 leu2Δ0 met15Δ0 ura3Δ0* | Research Genetics |
| BY4742 | *MATα his3Δ1 leu2Δ0 lys2Δ0 ura3Δ0* | Research Genetics |
| SPY122 | BY4741 *cth1::KanMX4 cth2::HisMX6* | (3) |
| YWO0607 | *MATa ura3 leu2-3,112 his3-11,15 Gal^+^* | D. H. Wolf |
| YWO0608 | YWO0607 *pre1-1* | D. H. Wolf |
| SPY131 | BY4741 *cth1::KanMX6* | (3) |
| SPY25 | BY4741 *cth2::KanMX4* | Research Genetics |
| SPY757 | BY4741 *grr1::KanMX4* | Research Genetics |
| SPY789 | BY4741 *cth2::hphB* | This study |
| yJD45-1 | BY4742 *grr1::NLS-mRFP-LEU2* | D. P. Toczyski |
| SPY790 | BY4742 *grr1::NLS-mRFP-LEU2 cth2::hphB* | This study |
| Y80 | *MATa can1-100 ade2-1 his3-11,15 leu2-3,112 trp1-1 ura3-1* | S. J. Elledge |
| Y552 | *Y80 skp1-11* | S. J. Elledge |
| *rbx1-1* | *MATa his3Δ200 ura3 leu2 trp1 lys2 can^R^ cyh^R^ rbx1::HIS3 pRS314-rbx1-1* | S. J. Elledge |
| AH326 | W303: *MATα ade2-1 can1-100 his3-11,15 leu2-3,112 trp1-1 ura3-1* | R. Loewith |
| SPY892 | AH326 *hrr25::KanMX4* | This study |
| SPY919  SPY970 | AH326 *hrr25::KanMX4 cth2::HisMX6 pdr5::KanMX4* | This study  Research Genetics |
