# Supplementary material for: Phosphorylation and Proteasome Recognition of the mRNA-Binding Protein Cth2 Facilitates Yeast Adaptation to Iron Deficiency
Source: mBio. 2018 Sep 18;9(5):e01694-18. doi: 10.1128/mBio.01694-18 (PMC6143738; doi:10.1128/mBio.01694-18)
Supplement: TABLE S2 [file mbo005184074st2.docx]

**Table S2. List of plasmids used in this study.**

| **Plasmid** | **Description** | **Source/Reference** |
| --- | --- | --- |
| pRS416 | CEN *URA3* | (29) |
| pSP480 | pRS416-Flag_2_-CTH1 | (4) |
| pSP414 | pRS416-Flag_2_-CTH2 | (3) |
| pSP421 | pRS426-Flag_2_-CTH2 | This study |
| pSP429 | pRS416-Flag_2_-CTH2-C190R | (12) |
| pSP851 | pRS416-Flag_2_-CTH2-S64A | This study |
| pSP852 | pRS416-Flag_2_-CTH2-S65A | This study |
| pSP853 | pRS416-Flag_2_-CTH2-S64A/S65A | This study |
| pSP898 | pRS416-Flag_2_-CTH2-S64A/S65A/C190R | This study |
| pSP883 | pRS416-Flag_2_-CTH2-S68A | This study |
| pSP884 | pRS416-Flag_2_-CTH2-S70A | This study |
| pSP856 | pRS416-Flag_2_-CTH2-S68A/S70A | This study |
| pSP899 | pRS416-Flag_2_-CTH2-S68A/S70A/C190R | This study |
| pSP892 | pRS416-Flag_2_-CTH2-S64A/S65A/S68A/S70A | This study |
| pSP875 | pYES2-655 | (14) |
| pSP878 | pYES2-GRR1ΔFbox-Flag | (14) |
| pSP886 | pRS413-Myc_2_-CTH2 | This study |
| pSP897 | pRS413-Myc_2_-CTH2-S64A/S65A | This study |
| pSP896 | pRS413-Myc_2_-CTH2-S68A/S70A | This study |
| pSP417 | pGEX-6P-1-CTH2 | This study |
| pSP907 | pGEX-6P-1-CTH2-S64A/S65A | This study |
| pSP908 | pGEX-6P-1-CTH2-S68A/S70A | This study |
| pSP909 | pGEX-6P-1-CTH2-S64A/S65A/S68A/S70A | This study |
| pCS180 | pGEX-6P-1-CTH2(62-74aa)-WT | This study |
| pCS183 | pGEX-6P-1-CTH2(62-74aa) -S64A/S65A/S68A/S70A | This study |
| AM4-1 | pGAL1-3HA-HRR25 | M. Cyert |
| AM8-2 | pGAL1-3HA-HRR25-K38A | M. Cyert |
