# Supplementary material for: Phosphorylation and Proteasome Recognition of the mRNA-Binding Protein Cth2 Facilitates Yeast Adaptation to Iron Deficiency
Source: mBio. 2018 Sep 18;9(5):e01694-18. doi: 10.1128/mBio.01694-18 (PMC6143738; doi:10.1128/mBio.01694-18)
Supplement: TABLE S3 [file mbo005184074st3.docx]

**Table S3. Oligonucleotides used for RT-qPCR in this study**

| **Name** | **Sequence (from 5’ to 3’)** |
| --- | --- |
| CTH2-qPCR-F | GCAGTTTCATTCTCTCCAC |
| CTH2-qPCR-R  SDH4-qPCR-F  SDH4-qPCR-R  HEM15-qPCR-F  HEM15-qPCR-R  RNR2-qPCR-F  RNR2-qPCR-R  RLI1-qPCR-F  RLI1-qPCR-R  ACT1-qPCR-F  ACT1-qPCR-R  PGK1-qPCR-F  PGK1-qPCR-R | TAGGTGCCGTGCTATTCAGG  GCACTCCCAATGATGCCTAC  AATGGAACGACGGACAAGG  CCAAAGTTGATGGCCTAATG  TATTCCGATTCCCCAATGAC  GGAGACATTGAGAGAGGAAAACAG  TGTGACGGTGAACTTGATGAG  GAGCCCCTGAATCTTTGTTG  CTTGGTCTGAAGGAGTTTGGA  TCGTTCCAATTTACGCTGGTT  CGGCCAAATCGATTCTCAA  AAGCGTGTCTTCATCAGAGTTG  CGTATCTTGGGTGGTGTTCC |
